# Diverse Honeydew-Consuming Fungal Communities Associated with Scale Insects

**DOI:** 10.1371/journal.pone.0070316

**Published:** 2013-07-26

**Authors:** Manpreet K. Dhami, Bevan S. Weir, Michael W. Taylor, Jacqueline R. Beggs

**Affiliations:** 1 Centre for Microbial Innovation, School of Biological Sciences, The University of Auckland, Auckland, New Zealand; 2 School of Biological Sciences, The University of Auckland, Auckland, New Zealand; 3 Landcare Research, Auckland, New Zealand; USDA-ARS, United States of America

## Abstract

Sooty mould fungi are ubiquitous, abundant consumers of insect-honeydew that have been little-studied. They form a complex of unrelated fungi that coexist and compete for honeydew, which is a chemically complex resource. In this study, we used scanning electron microscopy in combination with T-RFLP community profiling and ITS-based tag-pyrosequencing to extensively describe the sooty mould community associated with the honeydews of two ecologically important New Zealand coelostomidiid scale insects, *Coelostomidia wairoensis* and *Ultracoelostoma brittini*. We tested the influence of host plant on the community composition of associated sooty moulds, and undertook limited analyses to examine the influence of scale insect species and geographic location. We report here a previously unknown degree of fungal diversity present in this complex, with pyrosequencing detecting on average 243 operational taxonomic units across the different sooty mould samples. In contrast, T-RFLP detected only a total of 24 different “species” (unique peaks). Nevertheless, both techniques identified similar patterns of diversity suggesting that either method is appropriate for community profiling. The composition of the microbial community associated with individual scale insect species varied although the differences may in part reflect variation in host preference and site. Scanning electron microscopy visualised an intertwined mass of fungal hyphae and fruiting bodies in near-intact physical condition, but was unable to distinguish between the different fungal communities on a morphological level, highlighting the need for molecular research. The substantial diversity revealed for the first time by pyrosequencing and our inability to identify two-thirds of the diversity to further than the fungal division highlights the significant gap in our knowledge of these fungal groups. This study provides a first extensive look at the community diversity of the fungal community closely associated with the keystone insect-honeydew systems of New Zealand’s native forests and suggests there is much to learn about sooty mould communities.

## Introduction

Insect-excreted honeydew provides a high sugar source of nutrition, which is available to a range of organisms. In New Zealand, vast areas of native beech forests are infested with endemic scale insects (*Ultracoelostoma* spp., family: *Coelostomidiidae*) that produce up to 4500 kg dry weight/ha/year of honeydew [1]. A variety of organisms such as birds, insects, bats and lizards utilise this resource [2]. It is also consistently utilised by sooty mould fungi, which are the least-studied consumers of honeydew.

Sooty mould fungi are saprophytic facultative associates of honeydew-producing Hemiptera, particularly scale insects and aphids [3]. They form black-coloured colonies on surfaces that have received honeydew drip. These fungi include taxa from five families of *Ascomycota*, namely *Antennularielliaceae*, *Capnodiaceae*, *Chaetothyriaceae*, *Euantennariaceae* and *Metacapnodiaceae* (Order *Dothideales*) [4]. However, these identifications are largely based on morphological analysis of cultures and environmental samples and relatively few families considered as sooty moulds have been described using molecular methods. It is well known that only a small percentage of microscopic organisms can be cultured and sooty moulds have particularly rampant pleomorphic fruiting stages, further compromising visual identification. Thus, it is very likely that our current knowledge of the sooty mould fungal complex is incomplete.

Sooty mould fungi have a worldwide distribution, largely reflecting the distribution of the Hempitera which produce the honeydew, and their host plants [3]. These fungi have very low specificity to the host plant as some species may occur on up to 80 species of different host trees [4]. Their small spores allow virtually uninhibited dispersal to all environments [4]. Additionally, several species of sooty mould may coexist on the same host. For example, sooty mould fungi from seven genera are found in association with *Nothofagus* (beech) in New Zealand [5]. They are believed to derive their nutrients from coelostomidiid honeydew, which is a complex mixture of water-soluble carbohydrates such as sugars, sugar alcohols, water, free amino acids and proteins that varies among species [6]. This is believed to be due to differences in physiology of the scale insect species, rather than the influence of host tree or geographic location, as determined by previous work on honeydew compositional differences amongst these scale insect species [6]. Similar differences have been reported for the amino acid composition in honeydews of physiologically different aphids [7]. Fungi are able to metabolise various complex sugars, amino acids and sugar alcohols in the environment. Different species in the sooty mould complex may coexist by utilising different components of honeydew. If this were true, we would observe differences in the community composition of the sooty mould complexes observed on honeydews produced by different scale insect species.

In this study we extensively describe the taxonomic diversity of the sooty mould complex associated with honeydew from two species of New Zealand coelostomidiid scale insects, *Ultracoelostoma brittini* and *Coelostomidia wairoensis*, using molecular methods. This study represents the first such molecular analysis of an entire sooty mould community associated with insect-honeydew. We used terminal restriction fragment length polymorphism (T-RFLP) on the variable internal transcribed spacer (ITS) region of the fungal ribosomal operon to analyse the sooty mould community associated with *C. wairoensis* and *U. brittini*. A subset of these samples was further analysed using ITS-based tag-pyrosequencing (hereafter referred to as pyrosequencing) to assess in detail the community diversity and identify the taxa, which were present. We also utilised scanning electron microscopy in an attempt to disentangle the sooty mould complex using morphological characters such as fruiting bodies. We compared the sooty mould community compositional differences across factors such as the scale insect species and host plant, although the influence of host plant could only be robustly tested for a single species, *U. brittini*.

## Materials and Methods

### Sampling Design and Collection

**Figure 1:**
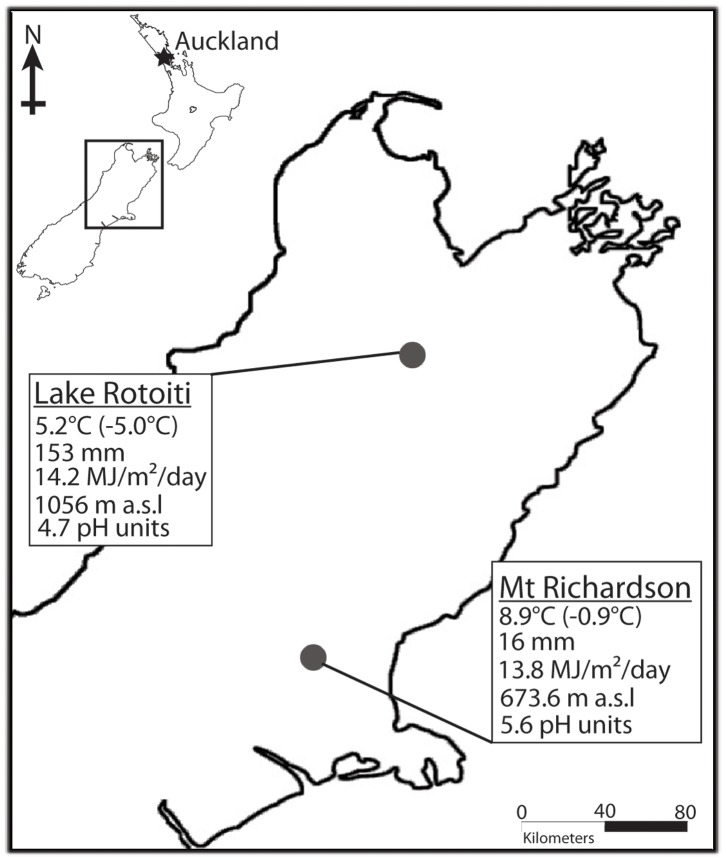
Map of upper half of South Island, New Zealand showing the two sites, Lake Rotoiti and Mt Richardson (Inset shows the map of New Zealand with the rectangle indicating the relative location of the magnified map of South Island). Below each site environmental data, namely, mean annual temperature (mean minimum temperature of the coldest month), mean annual rainfall, mean annual solar radiation, elevation and soil pH, are given. Data source: LRIS portal v. 1.0 Landcare Research (http://lris.scinfo.org.nz/) and Olliver & Co., via Koordinates (http://koordinates.com/#/maps/corax/).

The sooty mould samples were collected from multiple trees at three sites: Huia (Karamatura Valley), Auckland (−37.004051°, 174.556554°); Lake Rotoiti, Nelson (−41.832856°, 172.818879°) and Mt Richardson, Christchurch (–43.171771°, 172.218513°), New Zealand (Table 1). Reference samples from each of the localities are being kept in the New Zealand Fungal and Plant Disease Collection (PDD) as: PDD 102915, PDD 102916, PDD 102917 (http://scd.landcareresearch.co.nz/). For *Ultraceolostoma brittini*, samples were collected from two climatically distinct sites, namely Lake Rotoiti and Mt Richardson (approx. 160 km apart, Figure 1), to provide a comparison of site-influenced differences in sooty mould community composition. At Lake Rotoiti, samples were collected from black beech (*Nothofagus solandri* var. *solandri*) and red beech (*N. fusca*) to provide a comparison of sooty mould community composition across two host plant species (Table 1). At Mt Richardson, all samples were collected from black beech trees (Table 1). All samples from the Huia site were from kānuka (*Kunzea ericoides*) trees infested with *Coelostomidia wairoensis*. All necessary permits were obtained for the described field study. Collection permit to collect samples from Huia was provided by the Auckland Regional Council, New Zealand and for the Lake Rotoiti and Mt Richardson sites by the Department of Conservation, New Zealand.

**Table 1:** Sampling overview.

Scale species	*Ultracoelostoma brittini*			*Coelostomidia wairoensis*
Site	Lake Rotoiti		Mt Richardson	Huia
Host	Black beech	Red beech	Black beech	Kānuka
# of samples for T-RFLP	5	5	9	8
T-RFLP Sample IDs	LR1, LR4-7	LR2-3, LR8-10	MR2-10	MD1101, MD1103-1109
# of samples for pyrosequencing	3	2	3	4
Sample IDs for pyrosequencing	LR4, LR6-7	LR2 & LR8	MR8-10	MD1103, MD1107-1109

Sooty mould was collected from the surface of the tree trunk or branches using a sterile scalpel. Each slice of tissue was scraped off the surface and collected directly into a sterile 1.5 mL polypropylene tube containing 500 µL of absolute ethanol. The tubes were then transported to the laboratory and stored at –20°C until used.

### Scanning Electron Microscopy

Small strips (less than 10 mm × 5 mm) of bark were desiccated by air drying and then sputter coated thinly using a Polaron SC 7640 sputter coater with Pt (at 5–10 mA, 1.1 kV). The samples were then examined using a FEI Quanta 200 F Environmental Scanning Electron Microscope (ESEM) (USA). This ESEM uses a SiLi EDS detector with a super ultra-thin window and samples were processed at 10 kV and low vacuum (to reduce the structural alteration of any fruiting bodies present). Images were captured by the inbuilt EBSD detector at various magnifications.

### DNA Extraction and PCR Amplification

Total genomic DNA was extracted from the sooty mould samples using the ZyGEM Fungal/Bacterial DNA extraction kit (ZyGEM Corporation Ltd, Hamilton, New Zealand). 50–80 mg of tissue were physically comminuted by bead beating in a FastPrep® FP120 (Qbiogene Inc., Carlsbad, CA, USA) for 40 s at speed 5.5 ms^–1^. The DNA was precipitated, washed and eluted according to the manufacturer’s instructions. Purity and quantity of DNA were assessed using a NanoDrop 1000 spectrophotometer (Thermo Fisher Scientific GmBH,

Germany) and gel electrophoresis of a 5 µL aliquot on a 1% agarose gel containing 0.5 µg mL^−1^ ethidium bromide. The fungal ITS region was PCR-amplified from total genomic DNA using primers which target a wide range of fungal species (ITS-1F, ITS-4) [8], [9]. For T-RFLP analysis the forward primer (ITS-1F) was labelled with the dye 6-FAM and the reverse primer (ITS4) was labelled with the dye VIC. Each 25 µL PCR reaction mixture contained 10× buffer with 50 mM MgCl_2_ (2.5 µL), 25 pmol/µL primers (1 µL) each, 25 mmol DNTP mixture (2.5 µL), 1% BSA (1 µL), 5 µmol/µL Taq polymerase (0.3 µL), 1 µL DNA template and the remaining volume of molecular grade water [10]. Cycling parameters were an initial denaturing step (94°C, 4 min), 14 cycles of: denaturing (95°C, 35 s), annealing (55°C, 55 s) and extension (72°C, 45 s); followed by 15 cycles of: denaturing (95°C, 35 s), annealing (55°C, 55 s) and extension (72°C, 2 min); followed by 10 cycles of: denaturing (95°C, 35 s), annealing (55°C, 55 s) and extension (72°C, 3 min); and a final extension step (72°C, 10 min) [10].

### Restriction Digest

The ITS region amplicons were purified using the MinElute 96 UF PCR Purification Plates (Qiagen, USA). Aliquots of the purified amplicons were digested with the restriction enzymes CfoI and RsaI (Life Technologies, Carlsbad, CA, USA), as per the supplier’s instructions. The reaction mixture contained 5 µL of the purified PCR products, 0.2 µL of the endonuclease and 1 µL of the appropriate buffer, with molecular grade water bringing the volume up to 10 µL. The digestions were performed at 37°C for 3 h. Aliquots of the digests (0.5 µL) were mixed with 9 µL of HiDi formamide and 0.5 µL of internal size standard (MapMarker® 1000-ROX dye, BioVentures Inc., USA). The samples were denatured at 94°C for 10 min and then kept on ice until ready to run on the GeneScan mode of an automated ABI 3730*xl* Genetic Analyzer for 50 min. The ITS region fragments were size separated in relation to the internal size-standards. The fluorescently labelled 5′- and 3′ -terminal restriction fragment peak sizes were further analysed using GeneMapper v 4.1.

### Statistical Analysis of T-RFLP Profiles

A table of calibrated peak size and area was exported from GeneMapper, and imported into the statistical computing environment R [11] to run through the analysis package TRAMPR [12]. TRAMPR uses the peak-profiles obtained through combinations of primers and restriction endonucleases to match T-RFLP profiles of known organisms to those of mixtures of unknown profiles obtained from environmental samples. TRAMPR was used to determine unique patterns and classify them as “unknown knowns”, indicating the diversity of profile patterns present in each sample. The combined reference set of known peak-profiles (from fungal cultures associated with sooty mould, “knowns”) and “unknown knowns” was then used to compare the sample composition. All the identified (“knowns”) or unidentified (“unknown knowns”) profiles were clustered based on similarities in their peak-profiles using the inbuilt algorithm in TRAMPR. Alpha- and beta-diversity metrics were calculated using the package vegan 2.0–3 in R [13]. The summary of diversity matches for each sample against the combined “unknown knowns” and “knowns” database was then used to construct multi-dimensional scaling plots to visualise the differences between the samples. Furthermore, Adonis (F-test based on permuted sequential sums of squares) [14] and MRPP (multiple response permutation procedure) [15] implemented in the R environment (using the package vegan 2.0–3) were conducted to test for significant differences between the community profiles based on host tree for *U. brittini.* Exploratory analyses were conducted for geographic site-based differences and an overall comparison for the two scale insect species. Since these tests are non-parametric, statistical significance was computed using 999 permutations.

### ITS-based Tag-pyrosequencing

DNA extracts from 12 samples (Table 1) that displayed the highest richness in the T-RFLP analysis were amplified using the 454 primers for pyrosequencing of the ITS1 region (as above). Amplification primers were designed with FLX Titanium adapters and a multiplex identifier (MID) sequence directly on the forward and reverse ITS primer sequence (see Table S1 for details) (Roche Applied Sciences). For each 25 µL reaction, 1 µL of DNA template was used along with forward (A) and reverse (B) fusion primers (1 µL of 25 pmol/µL each), in a reaction mixture made up of 10X Buffer with 50 mM MgCl_2_ (2.5 µL), primers (1 µL) each, 25 mmol DNTP mixture (0.5 µL), 1% BSA (1 µL), 5 µmol/µL Taq polymerase (0.25 µL), 1 µL DNA template and the remaining volume of molecular grade water. PCR cycling conditions were an initial denaturing step (94°C, 3 min), 32 cycles of: denaturing (94°C, 30 s), annealing (52°C, 45 s) and extension (72°C, 1 min); followed by a final extension step (72°C, 8 min). PCR amplicons were cleaned and primer-dimers removed using the AgenCourt® AMPure® purification system (Beckman Coulter Inc., USA) as per the manufacturer’s instructions. The amplicons were quantified by fluorometry using the Quant-iT® PicoGreen dsDNA Assay kit (Invitrogen, USA), using the standard curve method as per the manufacturer’s instructions. Following quantification, amplicons were diluted and pooled as per the manufacturer’s instructions and emulsion PCR was conducted using kit A. The amplicon library was then sequenced on the GS Junior Titanium Genome Sequencer FLX System (Roche, NJ, USA) at Landcare Research, Auckland, New Zealand.

### ITS-based Tag-pyrosequencing Data Analysis

A total of 115,419 raw sequences were obtained. These sequences were then subjected to the Quantitative Insights Into Microbial Ecology (QIIME) pipeline for analysis [16]. The sequence library was first split by samples and quality filtered based on the quality scores for each sequence. Sequences with quality scores lower than 25, ambiguity, reads shorter than 100 bp or longer than 1000 bp, more than 2 mismatches in the primer, or a maximum homopolymer run exceeding 6, were removed. After the initial quality check they were subjected to stringent denoising, using flowgram clustering built into the QIIME pipeline [16]. Sequencing primers were also removed from the sequences in this step. The libraries were then combined and run through the “*Picking Operational Taxonomic Units (OTUs)”* workflow of the pipeline. The sequences were aligned using the MAFFT aligner [17] followed by filtering to remove positions that were all gaps. Chimera checking was performed using the BLAST-fragment method, where each sequence is split into three overlapping fragments and then BLAST-searched against the reference database (UNITE) and sequences returning different taxonomic identification for different fragments were removed from further analysis. OTUs were picked using the Uclust algorithm with 97% sequence similarities to create OTU tables. Taxonomy assignment of the sequences was done using the BLAST search algorithm [18] against the UNITE database [19] (Levels: Phylum 75% similarity; Class 80%; Order 85%; Family 90%; Genus 95%). RaxML [20] and FASTTREE [21] methods were used to build a phylogenetic tree of the sequences, and the data were summarised by taxa at all levels. Alpha diversity was calculated using the chao1 index and phylogenetic diversity (PD) estimates [22] by performing multiple rarefactions on the data (sampling depth of 4000 per sample). Following this, the rarefied OTU tables were used for jackknifed beta-diversity analysis. Weighted Unifrac (phylogenetically aware) [23] and the binary Jaccard’s coefficient [24] were used to calculate distance matrices. Principle coordinates analysis was performed on the distance matrices obtained from weighted Unifrac. Jaccard’s distance matrices were used for hierarchical UPGMA clustering. Adonis (F-test based on permuted sequential sums of squares) [14] and MRPP (multiple response permutation procedure) [15] were implemented in the R environment (using the package vegan 2.0–3) within the QIIME pipeline to analyse the distance matrices from weighted Unifrac and Jaccard’s and compare the sooty mould community differences between the two host tree species for *U. brittini*. Exploratory comparisons were conducted using the above specifications for geographic location-based differences for *U. brittini* and the overall comparison for the two scale insect species. Since these tests are non-parametric, statistical significance was computed using 999 permutations. The sequence data have been submitted to NCBI Sequence Read Archive under the accession number PRNJA200538.

## Results

### Morphological and Molecular Diversity Analysis of the Sooty Mould Complex

**Figure 2:**
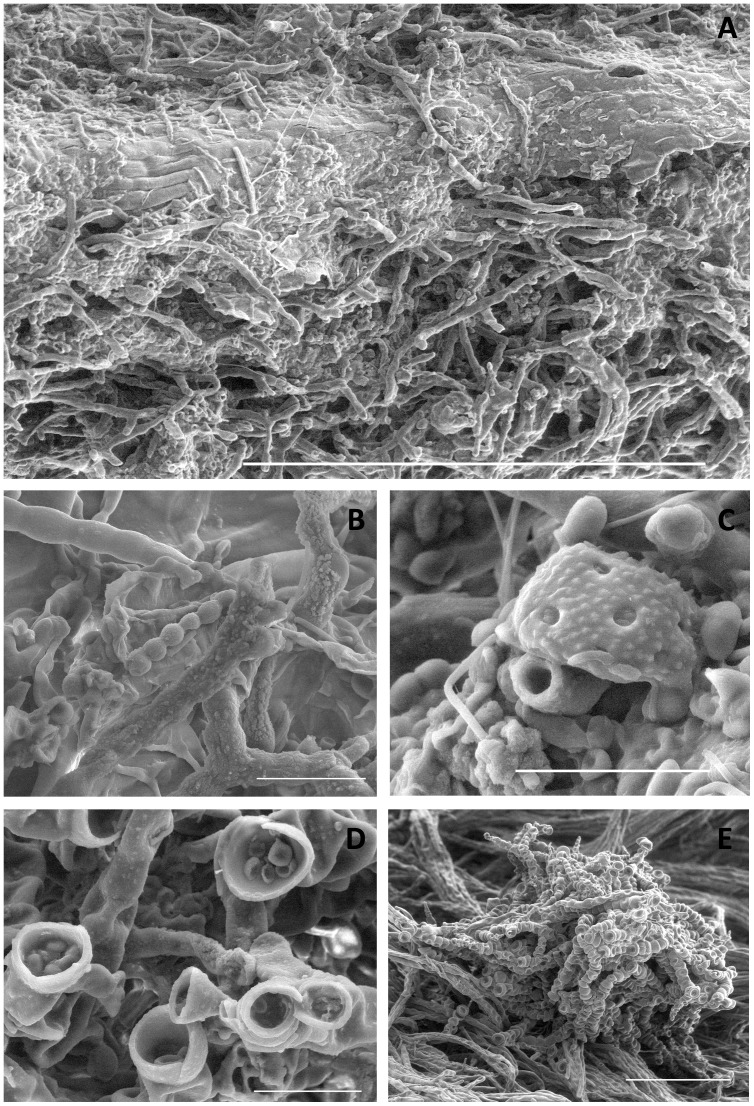
Sooty moulds associated with New Zealand coelostomidiid honeydew. Scanning electron micrographs of sooty mould from A, kānuka infested with *Coelostomidia wairoensis* (scale bar = 300 µm); B, kānuka infested with *C. wairoensis*, showing several types of smooth surface and rough surface (with projections) hyphae of different sizes and shapes (scale bar = 20 µm); C, kānuka infested with *C. wairoensis* showing a large rough surface spore-containing structure (scale bar = 20 µm); D, kānuka infested with *C. wairoensis* showing fruiting bodies (scale bar = 20 µm) and E, black beech infested with *Ultracoelostoma brittini* showing a cluster of fruiting bodies (scale bar = 250 µm).

Scanning electron microscopy (SEM) visualised the fungal hyphae and fruiting bodies in near-intact physical condition, with all parts of the fungi remaining turgid. A substantial range of fungal morphotypes was observed intertwined in the different samples from *Ultracoelostoma brittini*-infested beech trees and *Coelostomidia wairoensis*-infested kānuka (*Kunzea ericoides*) (Figure 2).

In order to extend upon the morphology-based observations described above, we employed two molecular techniques to elucidate the composition of the sooty mould communities. Terminal restriction fragment length polymorphism (T-RFLP) was used to describe overall fungal community structure, while amplicon pyrosequencing enabled us to determine the identities of these fungi.

**Figure 3:**
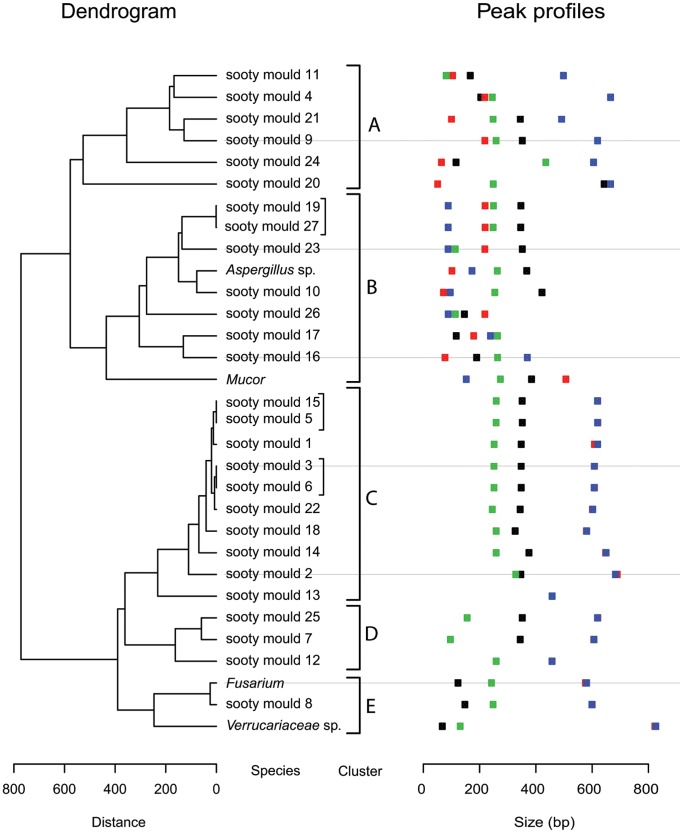
Unique “species” peak-profiles found in the sooty mould ITS-T-RFLP clustered on the basis of peak-profile similarities. Each peak-profile (far right) is composed of the end fragment lengths of restriction digests of the ITS-1 region using enzymes CfoI and RsaI (black = CfoI/ITS1F, red = CfoI/ITS4; green = RsaI/ITS1F, blue = RsaI/ITS4). Peak-profile clusters labelled A–E are based on distance-based similarity as predicted by the dendrogram (far left). Amongst the “species”, there are four cultured “knowns” namely *Aspergillus* sp., *Mucor*, *Fusarium* and *Verrucariaceae* sp. The remaining “knowns*”* are those identified from the unique peak-profiles *i.e.* “unknown knowns” denoted by sooty mould #.

Among the 27 sooty mould samples analysed (10 from Lake Rotoiti, 9 from Mt Richardson and 8 from Huia), a total of 24 unique T-RFLP peak-profiles were identified. These peak-profiles (hereafter referred to as “species”) clustered into five main groups based on the similarity of their restriction fragment lengths (Figure 3). The most speciose samples according to the T-RFLP analyses were from Mt Richardson (MR10) and Lake Rotoiti (LR3) (with 10 species each), and the least speciose sample was from Huia (MD1101) (1 species). Although sample replication was insufficient to allow formal statistical testing, exploratory diversity indices were calculated indicating that alpha and beta diversity were higher for sooty moulds associated with *U. brittini* than *C. wairoensis* (Table 2 and Figure S1).

**Table 2:** Summary alpha and beta diversity statistics based on the entire t-RFLP peak profile dataset of the sooty mould complex across the three different data partitions.

Factor	Variable	Alpha diversity	Beta diversity
Host	Black beech	6.928	1.453
	Red beech	4.75	1.316
	Kānuka	7	1.142
Site	L.Rotoiti	6.6	**1.576**
	Mt. Richardson	**7.333**	1.045
	Huia	4.75	1.316
Scale	*C. wairoensis*	4.75	1.316
	*U. brittini*	**6.947**	**1.59**

The most speciose of each group is in bold.

**Figure 4:**
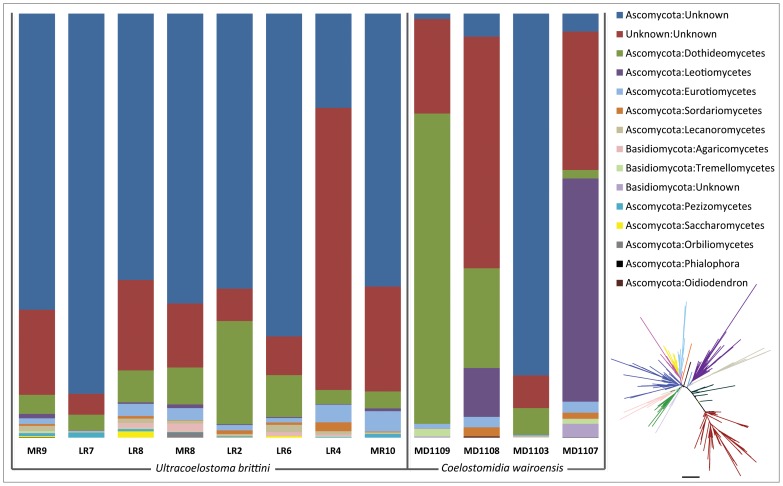
Top 15 most abundant fungal classes represented in the sooty mould ITS-based pyrosequencing dataset. The fungal classes are in order of abundance decreasing from top to bottom, with unknown fungi taking the top two spots, followed by several classes from *Ascomycota* and *Basidiomycota*. Representative phylogenetic tree coloured by most abundant fungal classes and others coloured dark teal. Scale bar = 1.0 substitutions/site. Each sample is labelled with Sample IDs from Table 1.

With the exception of the four cultured “knowns” fungi, whose T-RFLP peak-profiles appear to be discernible from the total community profiles (Figure 3), the other organisms detected by T-RFLP remain anonymous. For selected samples, we therefore amplified and sequenced the ITS-region of the fungal ribosomal RNA operon using tag-pyrosequencing, in order to identify the species present in the sooty mould community. A total of 43,150 sequences from 12 samples (Table 1) was analysed following denoising of the data. The mean number of sequences per sample was 3595 (range: 609–5248) and the total number of operational taxonomic units (OTUs) using a 97% similarity threshold was 2152. On average 243 different OTUs were present per sample (range: 85–449). Of all the sequences that were identified through BLAST against the UNITE database, up to 99.7% of reads were assigned to the kingdom fungi while 0.3% had no BLAST hits (and were removed from further analysis). Division *Ascomycota* was by far the most abundant taxon, accounting for 75.1% of assigned reads, followed by 23.9% belonging to “unknown fungi”, and a mere 1.1% of the reads belonging to *Basidiomycota*. Two sequences belonging to unknown *Chytridiomycota* (*incertae sedis*) were found in two of the Lake Rotoiti samples (LR4, LR8) (<0.01% each). Sequences affiliated with unknown *Ascomycota* dominated most of the samples, followed by unknown fungi samples. Among the fraction of *Ascomycota* sequences that could be assigned at class level, *Dothideomycetes* (that contain the majority of the described sooty mould families) was the most prevalent class, followed by *Leotiomycetes* and *Eurotiomycetes* (Figure 4). Besides *Ascomycota*, a few classes of *Basidiomycota* were also included in the 15 most abundant fungal classes (Figure 4). Rarefaction curves for each of the samples, calculated using expected phylogenetic diversity *vs.* observed phylogenetic diversity (PD estimate) and chao 1 estimate (for up to 4000 sequences/sample) plateaued, indicating sufficient sampling effort (Figure S2).

### Factors of Influence Driving Sooty Mould Composition

#### Scale insect host tree

**Figure 5:**
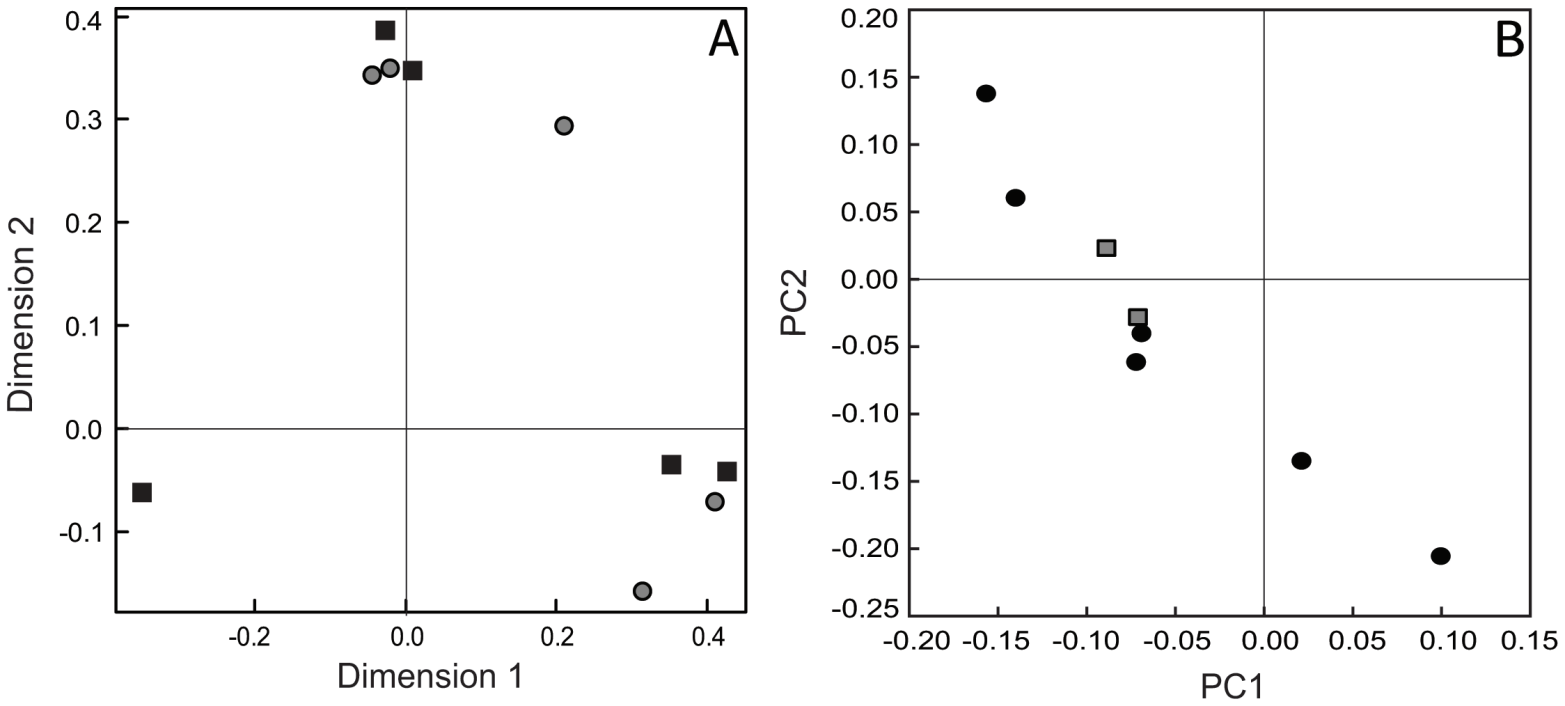
Host tree species influence on the composition of sooty mould community associated with *Ultracoelostoma brittini* honeydew. Plot A shows the multidimensional scaling plot based on T-RFLP peak-profiles using Bray-Curtis distances for samples from Lake Rotoiti with grey circles = red beech (n = 5) and black squares = black beech (n = 5); Plot B shows the principle coordinates analyses based on ITS-based pyrosequencing data using weighted Unifrac distances with grey squares = red beech (n = 2) and black circles = black beech (n = 6, sites pooled).

A main hypothesis of this study was that species of host tree would have a significant influence on the composition of associated sooty mould communities. To test this hypothesis we examined sooty moulds associated with honeydew produced by *Ultracoelostoma brittini* on two species of beech tree at the Lake Rotoiti site. Comparison of sooty mould composition (constructed by matching sample peak-profiles against the T-RFLP peak-profile database) of five samples taken from black beech *vs.* five from red beech revealed no distinct clustering of samples by host tree species when analysed by multidimensional scaling (Figure 5A). Moreover, statistical analysis using non-parametric tests (Adonis and MRPP) on the presence-absence matrices constructed from the composition data did not reveal significant differences in community composition (Table 3). Similar patterns were seen for the parallel comparison of the pyrosequencing dataset (principle coordinates analysis and non-parametric statistical testing, Figure 5B, Table 3), although a lesser number of samples (n*_total_* = 5) were available for comparison.

**Table 3:** Host influence on the sooty mould community composition using different molecular and analysis methods.

Molecular method	Replicates	Analysis		Statistic		Significance^2^
		Method	Distance matrix	Test statistic^1^	P-value	
T-RFLP	14, 9	Adonis	Binary	1.17	0.296	
		MRPP	Binary	0	0.414	
Pyrosequencing	6, 2	Adonis	Jaccard’s	0.99	0.573	
			w.Unifrac	0.75	0.588	
		MRPP	Jaccard’s	0.001	0.423	
			w.Unifrac	0.067	0.176	

Samples of sooty mould from black beech and red beech were compared.

1 Test Statistic: Adonis = F-test statistic; MRPP = chance corrected within-group agreement, A.

2 Significance codes: 0 ‘***’ 0.001 ‘**’ 0.01 ‘*’ 0.05.

#### Scale insect species

**Figure 6:**
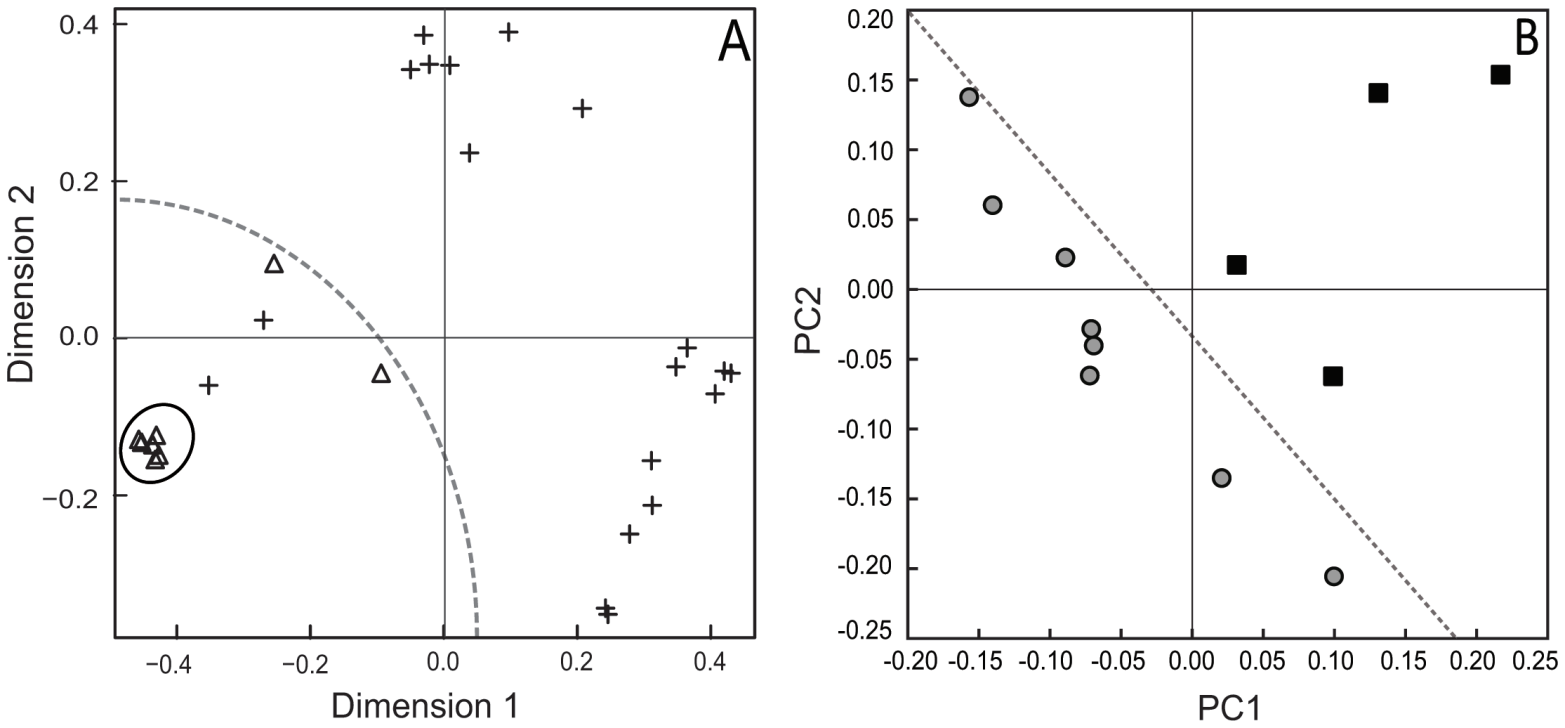
Overall pattern of scale species influence on the composition of sooty mould community associated with *Ultracoelostoma brittini* and *Coelostomidia wairoensis* honeydews. Plot A shows the multidimensional scaling plot based on T-RFLP peak-profiles using Bray-Curtis distances with “+” indicating sooty mould samples from *U. brittini* (n = 19, samples pooled from all sites and hosts) and open triangles for those from *C. wairoensis* (n = 8); Plot B shows the principle coordinates analyses based on ITS-based pyrosequencing data using weighted Unifrac distances with grey circles indicating samples from *U. brittini* (n = 8, samples pooled from all sites and hosts) and those from *C. wairoensis* indicated by black squares *Coelostomidia wairoensis* (n = 4).

Based on the lack of influence of host tree on the community composition of sooty moulds associated with *U. brittini*, all *U. brittini* samples (from L. Rotoiti and Mt Richardson) were compared with sooty mould samples associated with *Coelostomidia wairoensis* from a single host tree species (kānuka) at a single site Huia. For both the T-RFLP and ITS-based pyrosequencing datasets, marked differences in sooty mould community composition were evident across the two scale insect species (Figure 6). This pattern was further supported by non-parametric testing of the two datasets as described above (Table 4).

**Table 4:** Scale insect species influence on the sooty mould community composition using different molecular and analysis methods.

Molecular method	Replicates	Analysis		Statistic		Significance^2^
		Method	Distance matrix	Test statistic^1^	P-value	
T-RFLP	19, 8	Adonis	Binary	9.37	0.001	**
		MRPP	Binary	0.154	0.001	**
Pyrosequencing	8, 4	Adonis	Jaccard’s	2.04	0.004	**
			w.Unifrac	3.43	0.023	*
		MRPP	Jaccard’s	0.043	0.003	**
			w.Unifrac	0.084	0.026	*

Samples of sooty mould from *Ultracoelostoma brittini* and *Coelostomidia wairoensis* were compared.

1 Test Statistic: Adonis = F-test statistic; MRPP = chance corrected within-group agreement, A.

2 Significance codes: 0 ‘***’ 0.001 ‘**’ 0.01 ‘*’ 0.05.

#### Site

Geographic location may also influence the composition of sooty moulds due to differences in the local climate. We compared *Ultracoelostoma brittini* sooty mould samples from two locations with similar forest type (*Nothofagus*-podocarp forests) but different local climates (Figure 1). From one of the sites (Lake Rotoiti) we had samples from black beech and red beech, while at the second site (Mt Richardson), we had samples only from black beech. We compared black beech sooty mould samples from both sites (data not shown) and black beech sooty mould samples from Mt Richardson to black beech and red beech sooty mould samples from L. Rotoiti (since there was no detectable difference in the sooty mould community composition between the two host species at this site, Figure 5 and Table 3). Using the molecular and statistical approaches described above, we were unable to distinguish between samples from the different sites (Figure S3 and Table S2), despite their geographic separation and large climatic differences (Figure 1).

## Discussion

Sooty mould fungi are not only ubiquitous and abundant in the honeydew-scale insect system, but also highly diverse. Our study of sooty moulds associated with the ecologically important New Zealand scale insects, *Ultracoelostoma brittini* and *Coelostomidia wairoensis* indicates the diversity is at least one order of magnitude higher than previously thought and that two-thirds of the diversity could not be identified further than the fungal division, highlighting the significant knowledge gap that exists about this community of specialised consumers. Our more conservative approach estimated that the sooty mould community comprises 24 different “species” (unique peaks), whereas ITS-based tag-pyrosequencing yielded an average of >200 “species” (OTUs) from each sample. Although these diversity estimates differed substantially, both techniques identified very similar patterns of diversity across the hosts and sites of *U. brittini*. The sooty mould community complex is likely to be driven by the composition of the honeydew which is unique to each of the scale insect species, rather than host tree or site differences.

### Description of Sooty Moulds Associated with Honeydew

#### Morphological diversity of the sooty mould community

Although scanning electron microscopy revealed high morphological diversity amongst the sooty mould samples observed, the differences were not sufficient to reliably differentiate between sample types. None of the observed fruiting bodies could be visually morphology-matched with the drawings of New Zealand sooty moulds produced by Hughes [4], [25]–[30]. Overall, the study of fungal morphology in such a high diversity system does not provide the desired level of information, with previous attempts to identify sooty mould fungi using hyphal morphology suggesting that ‘characteristic’ surface projections of hyphae (Figure 2B) were often morphologically unstable [31].

#### Molecular diversity of the sooty mould community

Less than one-third (approx. 32%) of ITS sequences assigned to the most dominant division of fungi, *Ascomycota*, could be identified further, and these represented 48 families, within 33 orders and 11 classes. Finer-scale identification of remaining ascomycetes could not be achieved as the closest BLAST hits were to uncultured environmental samples (usually soil- or bark-associated). Even though 30 genera were identified (out of 265 individual OTUs belonging to *Ascomycota*), only a mere three species-level matches were found. This highlights the lack of taxonomically informative genetic data available for fungi and especially environmental fungi such as the sooty moulds. Most of the fungal genera that were identified were saprobes or lichenicolous with pigmented or blackened appearance. Some were plant pathogens, which is not surprising as these are the types of organisms that are usually well-studied and hence reflect a bias in the reference database against which the data were matched. Of the five sooty mould families described by Hughes [4], [26], [27], namely *Antennularielliaceae*, *Capnodiaceae*, *Chaetothyriaceae*, *Euantennariaceae* and *Metacapnodiaceae* (Class *Dothideomycetes*), only *Capnodiaceae* was detected in the sequence dataset, although nine other families belonging to the class *Dothideomycetes* were represented in the samples. The likely reason for this low identification rate is the absence of ITS-region sequence data for these sooty mould families. Recent molecular characterisation of sooty mould fungi within the family *Capnodiaceae* and related *Chaetothyriaceae* suggests that both these families contain unrelated taxa that belong to different classes but cannot be differentiated on the basis of morphology and growth habit [32], [33]. Sooty mould families from Orders *Chaetothyriales* (*Coccodiniaceae*) and *Pleosporales* (*Triposporipsidae*) [34] were not identified although other families from these orders were represented in this study. In addition, several previously unreported *Ascomycota* families were also detected in our study. Besides *Ascomycota*, 17 families within 12 orders of *Basidiomycota* were also identified. These have not been previously reported in association with scale insect honeydews. Within the basidiomycete class *Tremellomycetes*, we could identify three genera: *Sebacina*, *Cryptococcus* and *Tremella*. These are called jelly-fungi due to their gelatinous fruiting bodies [35]. *Tremella* are typically saprophytes of wood-rotting fungi [36] while *Sebacina* are usually ecto- or endomycorrhizal on forest trees [37]. *Cryptococcus* is generally a saprobe but some of the species are known to cause mammalian and human meningitis [38], [39]. Based on their ecological preferences, we believe that the basidiomycetes may be transients within the sooty mould complex, utilising the abundant honeydew-based physical environment composed mainly of *Ascomycota*-related fungi. Fungal spores from the basidiomycete fruiting bodies on the forest floor are likely to blow onto the tree trunks and branches and get trapped in the sooty mould mycelia. Similarly, the two sequences of unknown *Chytridiomycota* found in one of the samples are probably also a product of happenstance and are not ecologically relevant.

We were unable to identify a quarter of the fungal sequences, even down to Division level, due to lack of taxonomically detailed fungal sequences. Additional attempts to identify these sequences using the BLAST algorithm against the NCBI nucleotide database lead to low identity matches with uncultured fungi from environmental samples. These sequences may also represent novel fungal taxa.

### ITS-based T-RFLP *vs.* ITS-based 454 Pyrosequencing: which is the Better Diversity Estimator?

The ITS region is the best-studied gene region for fungi, currently with 320942 sequences available on UNITE and INSD (International Nucleotide Sequence Database). Although the ITS was used for both T-RFLP and pyrosequencing, the two molecular methods provided dramatically different estimates of the taxonomic richness of the sooty mould community. Realistically the true species richness is likely to fall between the two estimates of 24 and >200 species. The pronounced difference observed between the diversity estimates of the two methods can be explained by their technological differences. T-RFLP is based only on differences in restriction fragment sizes in order to separate different sequences. This relies on the fact that the ITS region is highly variable from species to species, but T-RFLP in general suffers from the invalid assumption that different microorganisms will always yield different fragment lengths. Additionally, not all sequences may be cleaved by a set of restriction enzymes, leading to long un-cleaved fragments which yield no information. Additionally, there is always the possibility that a single species with multiple copies of the ITS may produce multiple T-RFLP patterns, as seen in some arbuscular mycorrrhizal fungi [40]. Collectively, these limitations may lead to gross underestimation or sometimes even overestimation of diversity [41]. Whereas T-RFLP allowed rapid processing of a greater number of samples at minimal cost, ITS-based pyrosequencing provided a much larger amount of data per sample to process. However, often the large datasets from ITS-based pyrosequencing have a high proportion of low quality data and noise, so stringent measures must be applied to reduce errors and PCR biases [42]. For example, in this study over 115,419 sequence reads were obtained, of which only 43,150 were used for downstream analyses after denoising and chimera removal. Beyond these limitations, 454-pyrosequencing is likely to be a more comprehensive estimator of diversity, as it is based on direct sequence information. Careful examination of these sequence data may reduce the over-estimation of diversity, especially if a comprehensive reference database is available. However, even the more conservative T-RFLP technique indicates that our current knowledge of the sooty mould community complex is very limited.

### Does Species of Host Tree Influence Sooty Mould Community Composition?

Based on earlier studies within New Zealand and overseas [4], [26], [30], it has been suggested that sooty moulds do not have any host preference. However, it is possible that, at a nutritional level, host plants may influence scale insect honeydew composition, which could in turn influence the composition of sooty mould communities. This effect has been observed for some aphid species [43], however research in this area is largely lacking. For *Ultracoelostoma* spp. host plant influence on the composition of scale insect honeydew is minimal [6], perhaps explaining the apparent lack of influence of host tree species on the sooty mould community observed in this study. However, the narrow host preference of these scale insects prevents testing of this influence across phylogenetically distant host species: *U. brittini* attains large populations only on a few closely related beeches (*Nothofagus* spp.) [44], whereas the other species in this study, *Coelostomidia wairoensis*, feeds almost exclusively on a single host species (*Leptospermum scoparium*) [44]. This limits our ability to test the host influence on honeydew or sooty mould community composition. This lack of effect may therefore be limited to this system and not directly applicable to other honeydew-sooty mould systems where a diverse range of hosts may be utilised by some scale insect species.

### Does Species of Scale Insect Influence Sooty Mould Community Composition?

Of the several possible factors, it is likely that the honeydew composition of these two scale insect species is a crucial factor determining sooty mould community composition. The differences in honeydew composition have previously been attributed to differences in the physiology of these scale insects, especially in the presence of differing bacterial symbionts [6]. Both these scale insects are inhabited by a primary symbiont (*Bacteroidetes* sp.), potentially playing a role in their nutrition [45]. However, *C. wairoensis* is also inhabited by two other symbionts (*Wolbachia* sp., and an *Erwinia*-like symbiont) [45], that perhaps alter the nutrient pool, and hence influence the honeydew composition. In this study we found distinct sooty mould communities associated with the two scale insect species, according to both the T-RFLP and pyrosequencing datasets. We therefore propose that further research should be conducted on the influence that scale insect species and potentially their respective symbionts may be having on the composition of honeydew and therefore on the sooty mould consumers.

### Other Potential Determinants of Sooty Mould Community Composition

Despite the widespread distribution of sooty mould, one might expect that sooty mould fungi are influenced by the external environment. Lake Rotoiti is located in the subalpine zone with low annual temperatures, high annual rainfall and highly acidic soils (Figure 1). In contrast, Mt Richardson is situated in the low Canterbury hills characterised by moderate mean annual temperatures, an overall drier climate and less acidic soils (Figure 1). The presence of similar sooty mould communities at these two diverse sites suggests that environmental variables have a low level of influence on these fungi. However, it is important to note that only a few samples from two sites were included in this study and there may be underlying factors, which at this stage are not apparent, that confound these observed patterns. Nevertheless, earlier research indicates that sooty moulds are able to cope with microclimatic differences since they commonly form thick, spongy mat-like structures [4], so we think it is likely that geographical location has minimal influence on these ubiquitous microbial consumers of honeydew.

### Concluding Remarks

It is evident that sooty mould fungi are dependent on insect honeydew for sustenance [46], [47]. Our study evaluated the diversity of sooty moulds associated with scale insect honeydew using two fundamentally different community analysis techniques. Although T-RFLP identified an order of magnitude fewer species than pyrosequencing, both methods illustrate our lack of knowledge of taxonomic diversity of these fungi, since two-thirds of the diversity could not be identified further than the fungal division. We found distinct sooty mould communities among the scale insect species but detected no differences among the sooty mould communities associated with different host trees or site. There may be a range of factors responsible for the observed difference, for example, the strikingly different honeydew composition of these two scale insect species [6]. This study adds another example of differences in the observed consumer communities associated with these two scale insect species. These differences, the first of their kind reported, may be inherent to these unique New Zealand forest systems. Future work on the sooty mould community with greater sampling intensity may reveal additional levels of variation across scale insects, host trees, and habitats.

## Supporting Information

Figure S1 **Diversity indices for the sooty mould community diversity observed from all samples in the ITS-t-RFLP dataset.** H = Shannon index, simp = simpson index, invsimp = inverse simpson index, unbias.simp = unbiased simpson index and alpha = α coefficient of Fisher log series.
(TIF)

Figure S2 **Rarefaction curves showing the alpha diversity estimates Phylogenetic Diversity (PD) (top) and chao 1 (bottom).** Multiple rarefactions were performed using the method built in the QIIME pipeline. Briefly, OTU tables were rarefied with a minimum of 10 sequences/sample up to a maximum of 4000 sequences/sample, with stepsize = 10 sequences/sample and 10 iterations at each step.
(TIF)

Figure S3 **Site influence on the composition of sooty mould community associated with** ***Ultracoelostoma brittini*** **honeydew.** Plot A shows the multidimensional scaling plot based on T-RFLP peak-profiles using Bray-Curtis distances with grey squares = Mt Richardson (n = 9) and black circles = Lake Rotoiti (hosts pooled, n = 10); Plot B shows the principle coordinates analyses based on ITS-based pyrosequencing data using weighted Unifrac distances with open squares = Mt Richardson (n = 3) and open circles = Lake Rotoiti (n = 5).
(TIF)

Table S1 **List of ITS-based pyrosequencing samples and their respective FLX specific fusion tag-primers and their sequences.**
(DOCX)

Table S2 **Site influence on the sooty mould community composition using different molecular and analysis methods.** Samples of sooty mould associated with *Ultracoelostoma brittini* from Lake Rotoiti and Mt Richardson were compared.
(DOCX)
