# Supplementary figures and images for: Diverse Honeydew-Consuming Fungal Communities Associated with Scale Insects
Source: PLoS One. 2013 Jul 26;8(7):e70316. doi: 10.1371/journal.pone.0070316 (PMC3724830; doi:10.1371/journal.pone.0070316)

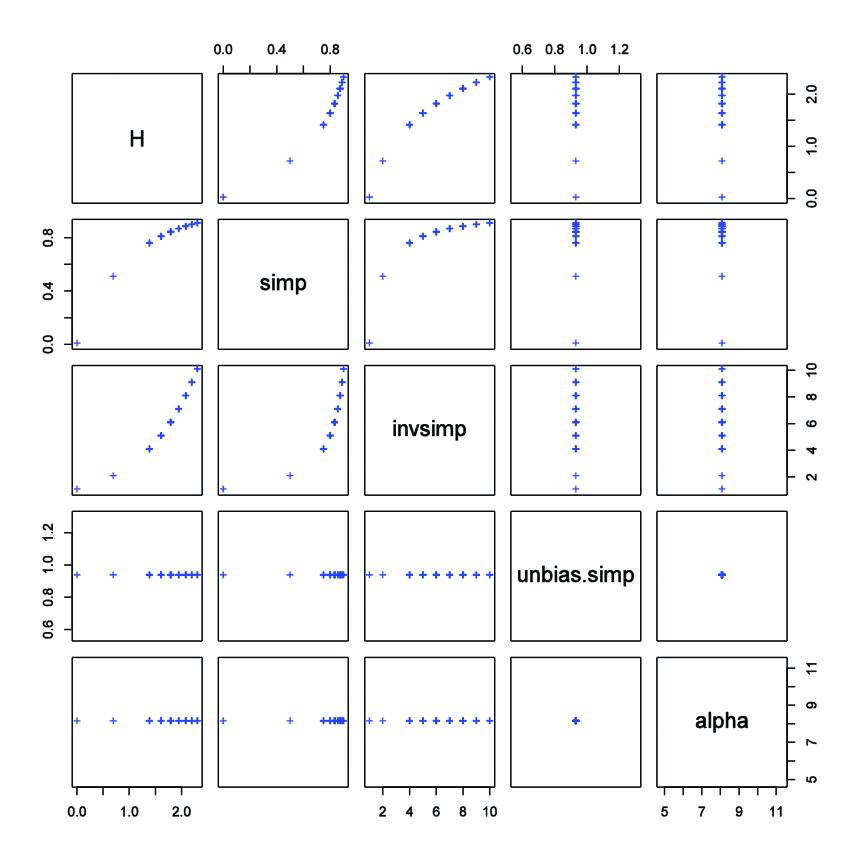

Supplement: Figure S1 — Diversity indices for the sooty mould community diversity observed from all samples in the ITS-t-RFLP dataset. H = Shannon index, simp = simpson index, invsimp = inverse simpson index, unbias.simp = unbiased simpson index and alpha = α coefficient of Fisher log series. (TIF) [file pone.0070316.s001.tif]

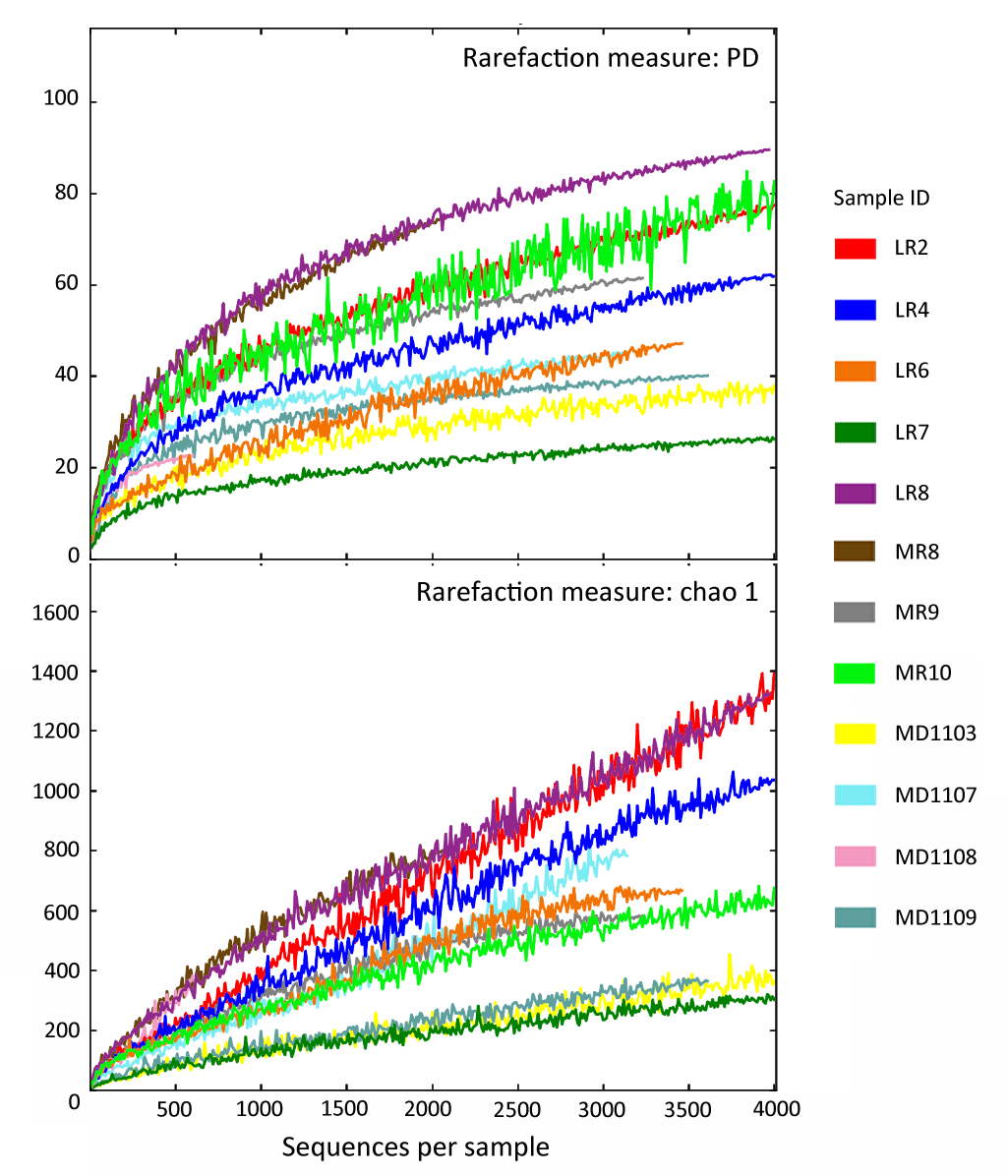

Supplement: Figure S2 — Rarefaction curves showing the alpha diversity estimates Phylogenetic Diversity (PD) (top) and chao 1 (bottom). Multiple rarefactions were performed using the method built in the QIIME pipeline. Briefly, OTU tables were rarefied with a minimum of 10 sequences/sample up to a maximum of 4000 sequences/sample, with stepsize = 10 sequences/sample and 10 iterations at each step. (TIF) [file pone.0070316.s002.tif]

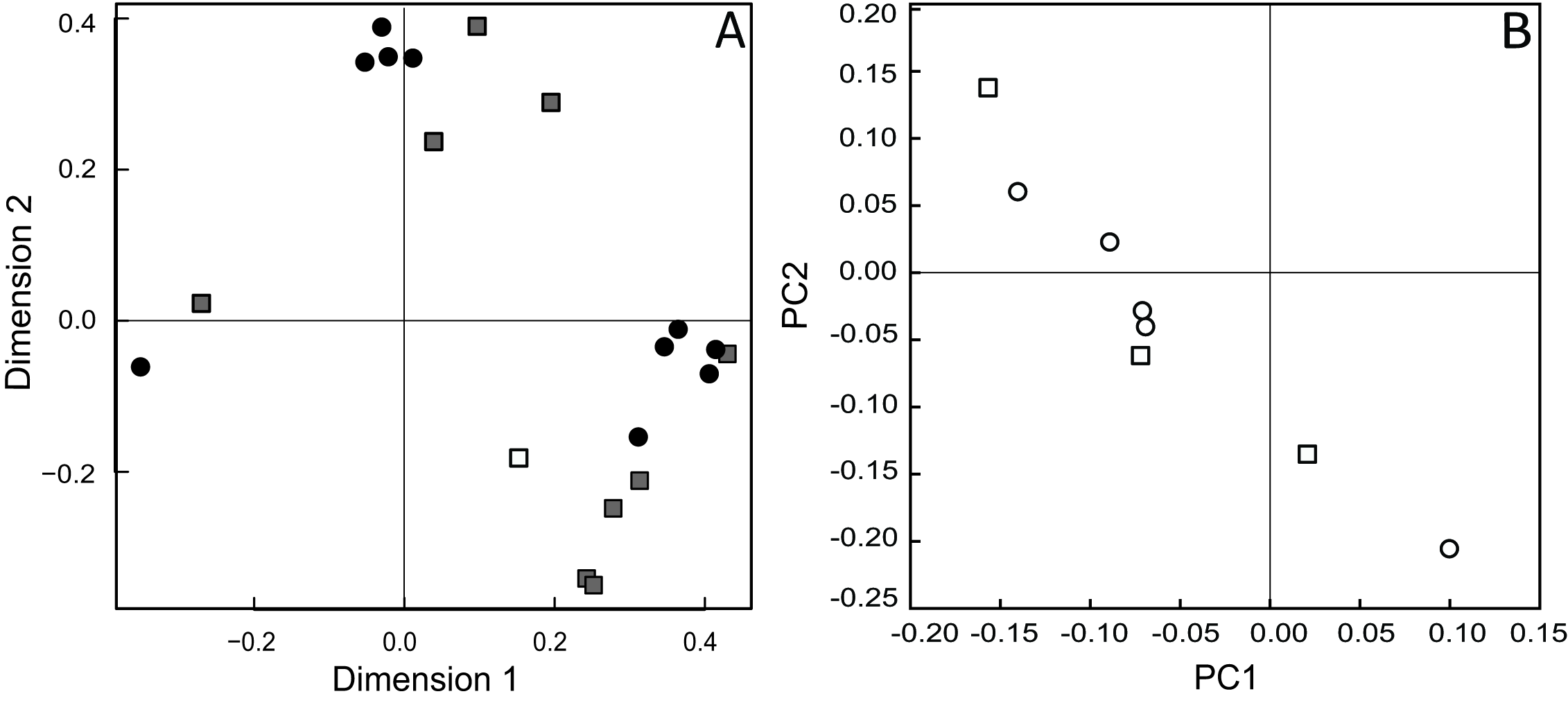

Supplement: Figure S3 — Site influence on the composition of sooty mould community associated with Ultracoelostoma brittini honeydew. Plot A shows the multidimensional scaling plot based on T-RFLP peak-profiles using Bray-Curtis distances with grey squares = Mt Richardson (n = 9) and black circles = Lake Rotoiti (hosts pooled, n = 10); Plot B shows the principle coordinates analyses based on ITS-based pyrosequencing data using weighted Unifrac distances with open squares = Mt Richardson (n = 3) and open circles = Lake Rotoiti (n = 5). (TIF) [file pone.0070316.s003.tif]
