# Supplementary material for: Diverse Honeydew-Consuming Fungal Communities Associated with Scale Insects
Source: PLoS One. 2013 Jul 26;8(7):e70316. doi: 10.1371/journal.pone.0070316 (PMC3724830; doi:10.1371/journal.pone.0070316)
Supplement: Table S1 — List of ITS-based pyrosequencing samples and their respective FLX specific fusion tag-primers and their sequences. (DOCX) [file pone.0070316.s004.docx]

| Sample ID | Primer-Tag combination | FLX Titanium Adaptors A or B (21bp)-Linker sequence (4bp)(red)-MID Adaptor sequence (10bp)-ITS1F or ITS4 primer sequence (20-22bp) (green) |
| --- | --- | --- |
| MR8 | ITS1F-MID1 | CGTATCGCCTCCCTCGCGCCATCAGACGAGTGCGTCTTGGTCATTTAGAGGAAGTAA |
| MR9 | ITS1F-MID2 | CGTATCGCCTCCCTCGCGCCATCAGACGCTCGACACTTGGTCATTTAGAGGAAGTAA |
| MR10 | ITS1F-MID3 | CGTATCGCCTCCCTCGCGCCATCAGAGACGCACTCCTTGGTCATTTAGAGGAAGTAA |
| MR6 | ITS1F-MID4 | CGTATCGCCTCCCTCGCGCCATCAGAGCACTGTAGCTTGGTCATTTAGAGGAAGTAA |
| MD1103 | ITS1F-MID5 | CGTATCGCCTCCCTCGCGCCATCAGATCAGACACGCTTGGTCATTTAGAGGAAGTAA |
| MD1107 | ITS1F-MID6 | CGTATCGCCTCCCTCGCGCCATCAGATATCGCGAGCTTGGTCATTTAGAGGAAGTAA |
| MD1108 | ITS1F-MID8 | CGTATCGCCTCCCTCGCGCCATCAGCTCGCGTGTCCTTGGTCATTTAGAGGAAGTAA |
| MD1109 | ITS1F-MID9 | CGTATCGCCTCCCTCGCGCCATCAGTAGTATCAGCCTTGGTCATTTAGAGGAAGTAA |
| LR2 | ITS1F-MID10 | CGTATCGCCTCCCTCGCGCCATCAGTCTCTATGCGCTTGGTCATTTAGAGGAAGTAA |
| LR4 | ITS1F-MID11 | CGTATCGCCTCCCTCGCGCCATCAGTGATACGTCTCTTGGTCATTTAGAGGAAGTAA |
| LR8 | ITS1F-MID13 | CGTATCGCCTCCCTCGCGCCATCAGCATAGTAGTGCTTGGTCATTTAGAGGAAGTAA |
| LR7 | ITS1F-MID14 | CGTATCGCCTCCCTCGCGCCATCAGCGAGAGATACCTTGGTCATTTAGAGGAAGTAA |
| MR8 | ITS4-MID1 | CTATGCGCCTTGCCAGCCCGCTCAGACGAGTGCGTTCCTCCGCTTATTGATATGC |
| MR9 | ITS4-MID2 | CTATGCGCCTTGCCAGCCCGCTCAGACGCTCGACATCCTCCGCTTATTGATATGC |
| MR10 | ITS4-MID3 | CTATGCGCCTTGCCAGCCCGCTCAGAGACGCACTCTCCTCCGCTTATTGATATGC |
| MR6 | ITS4-MID4 | CTATGCGCCTTGCCAGCCCGCTCAGAGCACTGTAGTCCTCCGCTTATTGATATGC |
| MD1103 | ITS4-MID5 | CTATGCGCCTTGCCAGCCCGCTCAGATCAGACACGTCCTCCGCTTATTGATATGC |
| MD1107 | ITS4-MID6 | CTATGCGCCTTGCCAGCCCGCTCAGATATCGCGAGTCCTCCGCTTATTGATATGC |
| LR7 | ITS4-MID7 | CTATGCGCCTTGCCAGCCCGCTCAGCGTGTCTCTATCCTCCGCTTATTGATATGC |
| MD1108 | ITS4-MID8 | CTATGCGCCTTGCCAGCCCGCTCAGCTCGCGTGTCTCCTCCGCTTATTGATATGC |
| MD1109 | ITS4-MID9 | CTATGCGCCTTGCCAGCCCGCTCAGTAGTATCAGCTCCTCCGCTTATTGATATGC |
| LR2 | ITS4-MID10 | CTATGCGCCTTGCCAGCCCGCTCAGTCTCTATGCGTCCTCCGCTTATTGATATGC |
| LR4 | ITS4-MID11 | CTATGCGCCTTGCCAGCCCGCTCAGTGATACGTCTTCCTCCGCTTATTGATATGC |
| LR8 | ITS4-MID13 | CTATGCGCCTTGCCAGCCCGCTCAGCATAGTAGTGTCCTCCGCTTATTGATATGC |

**Table S1** List of ITS-based pyrosequencing samples and their respective FLX specific fusion tag-primers and their sequences.
