# Supplementary material for: Diverse Honeydew-Consuming Fungal Communities Associated with Scale Insects
Source: PLoS One. 2013 Jul 26;8(7):e70316. doi: 10.1371/journal.pone.0070316 (PMC3724830; doi:10.1371/journal.pone.0070316)
Supplement: Table S2 — Site influence on the sooty mould community composition using different molecular and analysis methods. Samples of sooty mould associated with Ultracoelostoma brittini from Lake Rotoiti and Mt Richardson were compared. (DOCX) [file pone.0070316.s005.docx]

**Table S2** Site influence on the sooty mould community composition using different molecular and analysis methods. Samples of sooty mould associated with *Ultracoelostoma brittini* from Lake Rotoiti and Mt Richardson were compared.

| **Molecular method** | **Replicates** | | **Analysis** | | **Statistic** | | Significance^2^ |
| --- | --- | --- | --- | --- | --- | --- | --- |
|  |  |  | Method | Distance matrix | Test statistic^1^ | P-value |  |
| T-RFLP | | 10, 9 | Adonis | Binary | 2.17 | 0.068 |  |
|  |  |  | MRPP | Binary | 0.036 | 0.063 |  |
| Pyrosequencing | | 5, 3 | Adonis | Jaccard's | 1.15 | 0.119 |  |
|  |  |  |  | w.Unifrac | 1.09 | 0.413 |  |
|  |  |  | MRPP | Jaccard's | 0.015 | 0.06 |  |
|  |  |  |  | w.Unifrac | 0.021 | 0.35 |  |

^1^ Test Statistic: Adonis = F-test statistic;

MRPP = chance corrected within-group agreement, A

^2^ Significance codes: 0 ‘***’ 0.001 ‘**’ 0.01 ‘*’ 0.05
